# Mutations in Tyr^808^ reveal a potential auto-inhibitory mechanism of guanylate cyclase-B regulation

**DOI:** 10.1042/BSR20130025

**Published:** 2013-05-24

**Authors:** Takeshi Katafuchi

**Affiliations:** Department of Pharmacology, University of Texas Southwestern Medical Center at Dallas, TX, U.S.A.

**Keywords:** cGMP, C-type natriuretic peptide, guanylate cyclase-B, hyperactive mutation, ANP, atrial natriuretic peptide, CNP, C-type natriuretic peptide, DMEM, Dulbecco’s modified Eagle’s medium, FBS, fetal bovine serum, FGFR 3, fibroblast growth factor receptor 3, GC, guanylate cyclase, HNOBA, haem nitric oxide binding associated, IBMX, isobutylmethylxanthine, KHD, kinase-homology domain, LBD, ligand-binding domain, Myc-GC-B, Myc-tagged GC-B, PKC, protein kinase C, S1P, sphingosine-1-phosphate, VASP, vasodilator-stimulated phosphoprotein, WT, wild-type.

## Abstract

In this study, Tyr^808^ in GC-B (guanylate cyclase-B), a receptor of the CNP (C-type natriuretic peptide), has been shown to be a critical regulator of GC-B activity. In searching for phosphorylation sites that could account for suppression of GC-B activity by S1P (sphingosine-1-phosphate), mutations were introduced into several candidate serine/threonine and tyrosine residues. Although no novel phosphorylation sites that influenced the suppression of GC-B were identified, experiments revealed that mutations in Tyr^808^ markedly enhanced GC-B activity. CNP-stimulated activities of the Y808F and Y808A mutants were greater than 30-fold and 70-fold higher, respectively, than that of WT (wild-type) GC-B. The Y808E and Y808S mutants were constitutively active, expressing 270-fold higher activity without CNP stimulation than WT GC-B. Those mutations also influenced the sensitivity of GC-B to a variety of inhibitors, including S1P, Na_3_VO_4_ and PMA. Y808A, Y808E and Y808S mutations markedly weakened S1P- and Na_3_VO_4_-dependent suppression of GC-B activity, whereas Y808E and Y808S mutations rather elevated cGMP production. Tyr^808^ is conserved in all membrane-bound GCs and located in the niche domain showing sequence similarity to a partial fragment of the HNOBA (haem nitric oxide binding associated) domain, which is found in soluble GC and in bacterial haem-binding kinases. This finding provides new insight into the activation mechanism of GCs.

## INTRODUCTION

The membrane-bound GC (guanylate cyclase) family consists of five (human) and seven (rodent) members [1]. Among the human cyclases, GC-A is a receptor for ANP (atrial natriuretic peptide) and BNP (brain natriuretic peptides), GC-B is a receptor for CNP (C-type natriuretic peptide) and GC-C is a receptor for guanylins (guanylin and uroguanylin). GC-A is expressed primarily in the cardiovascular system, where it regulates vascular tone and body fluid level [1]. GC-B is expressed in a variety of tissues, including chondrocytes, female reproductive organs, the CNS (central nervous system) and fibroblasts, and is believed to regulate morphogenesis [2–4]. GC-C is mainly expressed in the digestive system and regulates ion transport and crypt growth [5]. Little is known about the extracellular activating mechanisms of GC-E and GC-F, which are specifically expressed in retinal and olfactory cells, respectively, and regulate cGMP levels that control sensory processes [6,7].

Membrane-bound GCs consist of three major functional domains: an extracellular LBD (ligand-binding domain), an intracellular KHD (kinase-homology domain) and a GC catalytic domain [1]. Several lines of biochemical evidence [8–11], including the elucidation of the three-dimensional structure of its LBD [12,13], indicate that GC-A forms at least a homodimer. Upon binding to ligand, the two subunits of the LBD dimer re-orient their positions with respect to each other, giving rise to rotation of each of the juxtamembrane domains, presumably causing structural alteration(s) of the catalytic domains that elicit GC activity [12]. As other GCs show high sequence similarity to GC-A, they are likely to be activated by a similar mechanism. The KHD has slight but significant amino acid sequence identity to the tyrosine kinase domain of PDGF (platelet-derived growth factor) [14]. However, the KHD is apparently unable to catalyse protein phosphorylation, but binds to ATP in the presence of Mg^2+^ to stabilize GC activity [15].

CNP-induced GC activity is suppressed in cells on treatment of cells with a wide variety of agents, including S1P (sphingosine-1-phosphate) [16,17], LPA (lysophosphatidic acid) [18], growth factors [19], peptides [20,21], the Ca^2+^ ionophore, ionomycin [22], the PKC (protein kinase C) activator, PMA [23,24] and the tyrosine phosphatase inhibitor, Na_3_VO_4_ [19]. Potter's group have identified six potential phosphorylation sites clustered between residues 513 and 529 in the juxtamembrane region of the KHD [25], and demonstrated that GC-B undergoes PKC- and Ca^2+^-dependent dephosphorylation upon treatment with S1P, PMA and ionomycin [16–18,26]. However, mutation of the phosphorylatable residues with phosphomimetic residues such as glutamic acid had insignificant effects on reduction of activity by S1P and ionomycin treatment [24], pointing to the existence of inhibitory mechanisms that do not involve dephosphorylation of these six residues. Based on these observations, I hypothesized that GC-B contains previously unidentified regulatory phosphorylation sites. A database search revealed 14 potential serine/threonine and two tyrosine phosphorylation sites, which I mutated to alanine and phenylalanine, respectively. Although the majority of these mutations had negligible effects on GC-B, the Y808F mutant expressed remarkably higher activity than WT (wild-type) GC-B. Further analysis of this and other mutations of residue Tyr^808^ highlighted its importance in the regulation of GC-B activity. The characteristics of these Tyr^808^ mutants, which may prove useful in future structural, mechanistic and pharmacological investigations, are examined in this study.

## EXPERIMENTAL

### Construction of GC-B mutants

Mutations were introduced into WT Myc-GC-B (Myc-tagged GC-B) cDNA by PCR using primers shown in Supplementary Table S1 (at http://www.bioscirep.org/bsr/033/bsr033e039add.htm). PCR was carried out in a reaction containing 0.2 mM dNTP, 1× reaction buffer, 50 units/ml Pfu polymerase and Myc-GC-B cDNA (1 ng/reaction) with a sense primer (GCB1688s) 5′-AAGCTGATGCTGGAGAAGGA-3′ and an antisense primer for each of the mutations in Table S1, or an antisense primer (GCBmyca) 5′-GCGGCCGCTCACAGAT-CCTCTTCTGAGATGAGT-3′ and a sense primer for the same mutation in Table S1. The reactions were mixed, and PCR was performed again using GCB1688s and GCBmyca primers. The amplified fragment was digested with NotI and NheI, ligated with N-terminal HindIII–NheI fragment of GC-B, and introduced into HindIII–NotI-digested pcDNA3.1 mammalian expression vector.

### Measurement of intracellular cGMP level

HeLa cells were cultured in DMEM (Dulbecco's modified Eagle's medium) containing 10% (v/v) heat-inactivated FBS (fetal bovine serum) and antibiotics (100 units/ml penicillin and 100 μg/ml streptomycin). Cells were seeded onto poly-d-lysine-coated 12- or 24-well plates 8–24 h prior to transfection. WT or mutant Myc-GC-B cDNAs were transfected into HeLa cells using Lipofectamine™ 2000 according to the manufacturer's protocol. The medium was replaced with DMEM containing 0.5% FBS 6–12 h after transfection, and cells were further cultured for 24 h. The medium was replaced again with serum-free DMEM containing 20 mM Hepes (pH 7.3) and incubated for 2 h. Following addition of IBMX (isobutylmethylxanthine) and incubation for 15 min at 37°C, CNP was added to each well, and cells were incubated for 5 min at 37°C to measure maximal intracellular cGMP accumulation [27]. To evaluate the effect of S1P, PMA and Na_3_VO_4_ on GC-B activity, each reagent was added 30 min prior to addition of IBMX. The incubation medium was them removed by aspiration, and 95% (v/v) ethanol was added to each well. Following one freeze-thaw cycle, the solution in each well was transferred into a test tube, and the solvent was removed using a Speedvac model SC200. The resulting pellet was dissolved in serum-free DMEM containing 20 mM Hepes (pH 7.3) and succinic solution (1.4.-Dioxiane:triethylamine:acetic anhydride=20:5:1) was added to the dissolved samples. Following a 30 min incubation at room temperature (22°C), the solvent was evaporated using a Speedvac, and the resulting pellet was dissolved in 90 mM CH_3_COONa (pH 6.2). An aliquot of each sample was used for measurement of cGMP by RIA as described previously [19].

### Measurement of GC activity in the plasma membrane

WT or mutant GC-B was expressed in HeLa cells as described above. Medium was replaced with DMEM containing 0.5% FBS 26 h prior to harvesting and again with serum-free DMEM 2 h prior to harvesting. The cells were washed twice with ice-cold PBS, and harvested with a homogenization buffer containing 50 mM Tris (pH 7.5), 1 mM EDTA and 10 μg/ml each of *Nα*-*p*-tosyl-*L*-lysine chloromethyl ester, *Nα*-*p*-tosyl-*L*-arginine methyl ester, *Nα*-*p*-tosyl-*L*-lysine chloromethyl ketone, leupeptin, pepstatin A, 1 mM PMSF and 1× concentration of phosphatase inhibitor cocktail 1 and 2 (Sigma). Harvested cells were then centrifuged for 5 min at 21000 ***g***, sonicated with the homogenization buffer, and centrifuged again for 15 min at 21000 ***g***. The pellet was washed once with homogenization buffer and sonicated again. Following measurement of protein concentration using the BCA (bicinchoninic acid) protein assay kit (Pierce), the homogenate was aliquoted to obtain a protein amount of 50 μg/tube. Reaction was allowed to proceed for 5 min at 37°C in 100 μl of reaction buffer (50 mM Tris/HC1, pH 7.5, containing 0.5 mM IBMX, 0.1% BSA, 15 mM creatine phosphate, 3.5 units of creatine kinase, 1 mM GTP, 1 mM ATP, 4 mM MgC1_2_ and 1 μM CNP). To measure Triton X-100/Mn^2+^-dependent GC activity, ATP and MgC1_2_ were replaced by 1% (v/v) Triton X-100 and 4 mM MnC1_2_. Reactions were terminated by addition of 25 μl of 1 N HClO_4_. Following centrifugation for 5 min at 21000 ***g***, each supernatant was used to determine cGMP concentration as described above.

### Immunoblotting of Myc-GC-B mutants

HeLa cells expressing Myc-GC-B or its mutants were homogenized in buffer containing 62.5 mM Tris (pH 6.8), 5% (w/v) SDS, 0.025% Bromophenol Blue and 2% (v/v) 2-mercaptoethanol. The homogenates were boiled for 3 min, then vortex-mixed vigorously to reduce viscosity. Aliquots of the homogenates were resolved on an SDS/6% PAGE, and then blotted onto a nitrocellulose membrane. WT and mutant GC-Bs were recognized using monoclonal anti-myc antibody 9E10 (National Cell Culture Center).

### Quantification of GC-B phosphorylation

Myc-GC-B or Y808E mutant cDNA was transfected into HeLa cells grown in 6-well plates, and the cells were cultured in the growth medium overnight. After 24 h culture in DMEM containing 0.5% FBS, cells were labelled with serum- and phosphate-free DMEM containing 0.1 mCi/ml [^32^P]orthophosphate for 4 h. HeLa cells expressing Myc-GC-B were homogenized on ice with RIPA buffer [50 mM Tris/HCl (pH 8.0), 150 mM NaCl, 1% (v/v) NP-40, 0.5% deoxycholate, 0.05% SDS and protease and protein phosphatase inhibitors as described above], and the homogenates were centrifuged for 10 min at 21000 ***g*** at 4°C. The resulting supernatants were transferred to a tube containing mouse anti-myc antibody (5 μg/ml) and mixed for 4 h at 4°C. Protein G-Sepharose was then added to each tube and further mixed for 2 h at 4°C. The Protein-G-Sepharose-anti-myc antibody complex was washed five times with RIPA buffer, and the final pellet was mixed with 2× SDS/PAGE sample buffer (Bio-Rad) containing 5% (v/v) 2-mercaptoethanol. Samples were boiled for 3 min, aliquots of the homogenate were separated by SDS/PAGE (6% polyacrylamide gel), and then blotted on to a nitrocellulose membrane. Phosphorylated WT Myc-GC-B and Y808E bands were detected by autoradiography, and WT Myc-GC-B and Y808E proteins were detected using anti-myc antibody and the ECL system (GE).

## RESULTS

### Mutations of Tyr^808^ enhance GC-B activity

**Figure 1:**
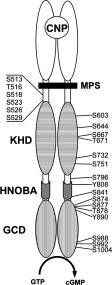
Schematic representation of the structure of GC-B and location of potential phosphorylation sites All verified juxtamembrane phosphorylation sites are shown on the left, and potential serine/threonine and tyrosine phosphorylation sites predicted by the NetPhos 2.0 program are shown on the right. Each potential phosphorylation site was mutated to alanine (serine/threonine) or phenylalanine (tyrosine) using primers shown in Table S1. Blank ovals and filled bar show LBD and plasma membrane, respectively. CNP, C-type natriuretic peptide; MPS, multiple phosphorylation site; KHD, kinase-homology domain; HNOBA, the domain highly homologous to haem nitric oxide binding associated domain; and GCD, guanylyl cyclase (catalytic) domain.

**Figure 2:**
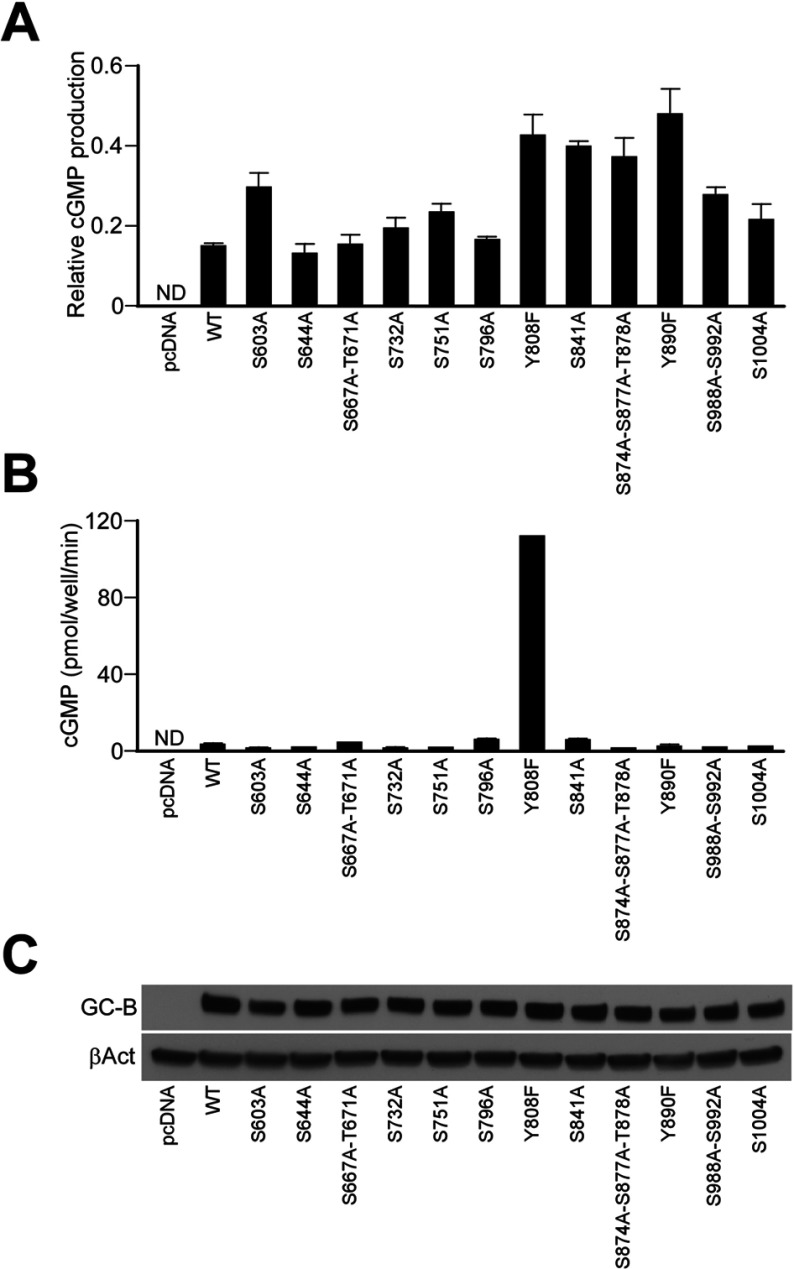
cGMP production in HeLa cells expressing WT Myc-GC-B and its mutants (**A**) Effect of mutations on the inhibition of GC-B activity by S1P. WT and mutant forms of Myc-GC-B were expressed in HeLa cells preincubated in medium with or without 100 nM S1P for 30 min, and then stimulated with 20 nM CNP for 5 min. Bars represent the ratios of amount of cGMP produced in the presence against the absence of S1P. (**B**) CNP-stimulated cGMP production in HeLa cells expressing WT and mutant forms of Myc-GC-B. Cells were treated with 0.1 μM CNP for 5 min prior to measurement of cGMP production. (**C**) Protein levels of WT and mutant Myc-GC-B in HeLa cell extract detected by immunoblotting with anti-myc antibody. βAct (β-actin) was used as a loading and transfer control. Each bar in panels (**A**) and (**B**) represents the means±S.E.M., *n*=3.

As stated, there are six phosphorylation sites in the juxtamembrane portion of the KHD (designated MPS in Figure 1), but dephosphorylation of these sites cannot fully account for the suppression of GC-B activity by S1P or other inhibitors. Therefore I postulated the existence of hitherto unidentified phosphorylation sites that could be susceptible to regulation by GC-B inhibitors. To identify these hypothetical phosphorylation sites in GC-B, I employed the NetPhos 2.0 phosphorylation site prediction program [28] (http://www.cbs.dtu.dk/services/NetPhos/), and selected residues having scores greater than 0.800 for mutation. Based on this search, I identified 14 potential serine/threonine phosphorylation sites and two potential tyrosine phosphorylation sites which had not previously been examined (Figure 1). I replaced these serine/threonine and tyrosine residues with alanine and phenylalanine, respectively, expressed the mutants in HeLa cells, and measured CNP-stimulated cGMP production. As shown in Figure 2A, none of the mutations eliminated S1P-dependent inhibition of cyclase activity, though several of the mutants were less susceptible to inhibition than WT GC-B.

**Figure 3:**
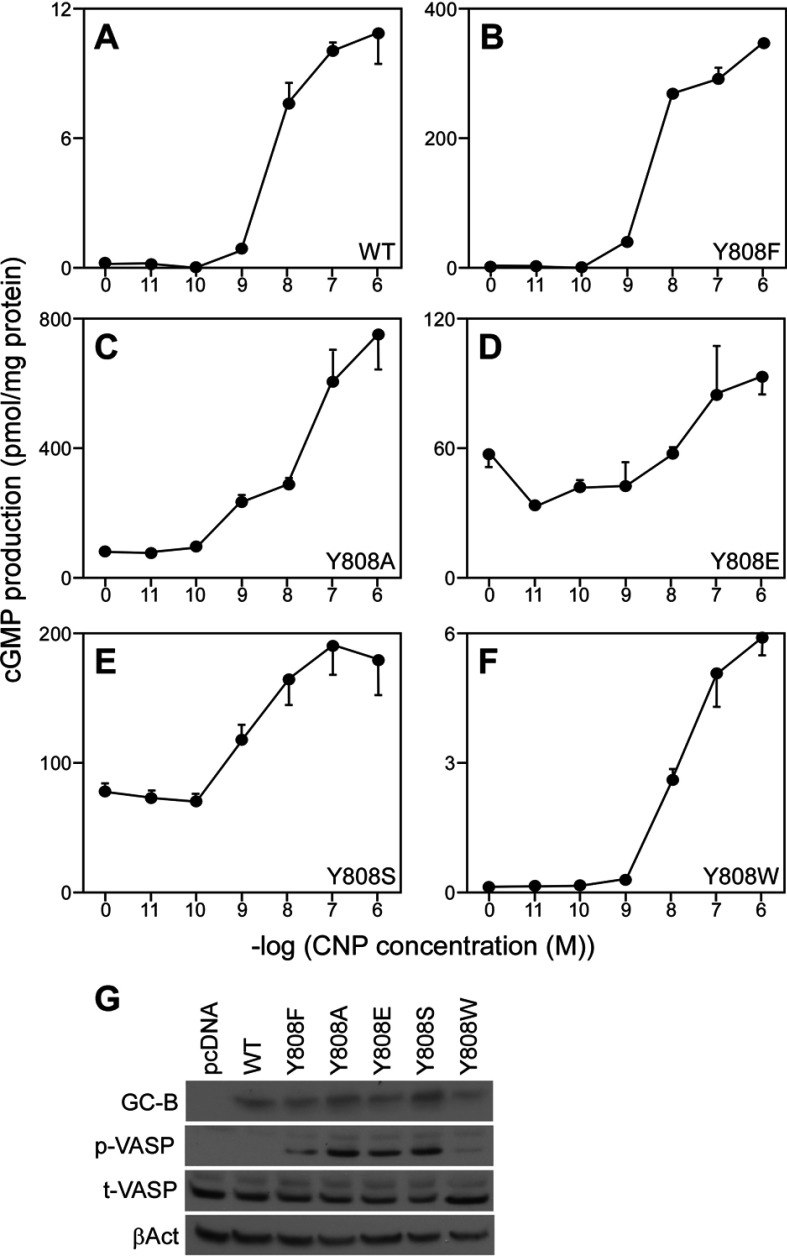
Dose–response elevation of cGMP production by WT Myc-GC-B and Tyr^808^ mutants HeLa cells expressing WT Myc-GC-B (**A**) or Tyr^808^ mutants, such as Y808F (**B**), Y808A (**C**), Y808E (**D**), Y808S (**E**) and Y808W (**F**) were stimulated with indicated concentrations of CNP for 5 min, and cGMP production was measured. Data represent means±S.E.M., *n*=3. (**G**) Levels of Myc-tagged WT GC-B and its Tyr^808^ mutants (GC-B), P-VASP (phosphorylated VASP), t-VASP (total VASP) and βAct (β-Actin) in HeLa cells determined by immunoblotting.

Although my mutational analysis indicated that S1P-mediated suppression of GC-B activity cannot be explained by a change in phosphorylation of any of the candidate phosphorylation sites (at least individually), I found that cells expressing the Y808F mutant produced more than 30-fold higher levels of cGMP than cells expressing WT GC-B upon stimulation with 0.1 μM CNP (Figure 2B), despite lower levels of expression of the mutant cyclase (Figure 2C). Phosphorylation of Tyr^808^ did not contribute to this effect, as neither WT GC-B nor the Y808F mutant was recognized by the 4G10 anti-phosphotyrosine antibody (not shown). To further analyse the significance of Tyr^808^ for GC-B activity, I substituted this residue with amino acids having different chemical characteristics. The effect of residue volume was examined by substituting Tyr^808^ with smaller (alanine) or larger (tryptophan) residues (residue volumes of alanine, phenylalanine, tyrosine and tryptophan are 67, 135, 141 and 186 Å^3^, respectively [29]). The importance of hydrophilicity was examined by replacing Tyr^808^ with glutamic acid and serine. I expressed these mutants in HeLa cells to measure CNP-dependent cGMP production. Like the Y808F mutant, the Y808A, Y808E and Y808S mutants exhibited markedly higher GC activity than did WT GC-B (Figure 3). Upon stimulation with 1 μM CNP, cells expressing the Y808A, Y808E and Y808S mutants produced more than 70- (750 pmol/mg protein), 8- (92.9 pmol/mg protein) and 16-fold (178.9 pmol/mg protein) higher levels of cGMP than WT (10.7 pmol/mg protein), respectively (Figures 3A, 3C, 3D and 3E). Moreover, these three mutants were constitutively active, generating cGMP in cells 370-fold (78.3 pmol/mg protein, Y808A), 270-fold (56.8 pmol/mg protein, Y808E) and 370-fold (78.1 pmol/mg protein, Y808S) higher than WT (0.2 pmol/mg protein) (Figures 3A, 3C, 3D and 3E), even without CNP stimulation. Interestingly, the level of VASP (phosphorylated vasodilator-stimulated phosphoprotein), a well-characterized substrate of cGMP-dependent kinase [30], is higher in the cells expressing Y808A, Y808E and Y808S mutants than in cells expressing WT-GC-B even without CNP stimulation (Figure 3G). These data indicate that the cGMP production is elevated in intact cells expressing the mutants. Despite the enhancement of catalytic potential by these Tyr^808^ mutations, the EC_50_s of WT and mutant GC-Bs were similar, indicating that the mutations did not alter ligand–receptor interactions. The EC_50_ of the single mutant that displayed lower than WT activity, Y808W, was also unchanged. In summary, GC-B mutants containing smaller and more hydrophilic residues than Tyr^808^ expressed remarkably higher GC activity than WT.

### Hyperactive mutants are less susceptible to GC-B inhibitors

**Figure 4:**
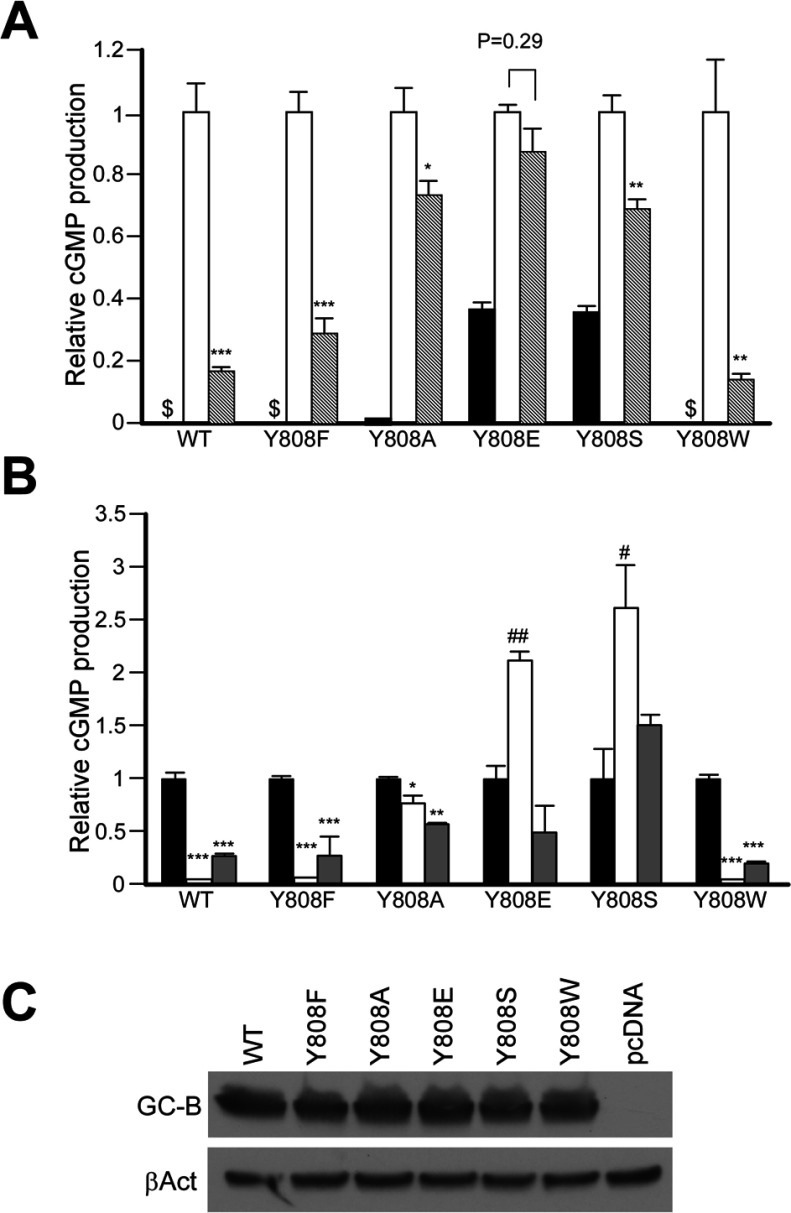
Effect of S1P, PMA and orthovanadate on cGMP production in WT Myc-GC-B and Tyr^808^ mutants (**A**) HeLa cells transiently expressing Myc-tagged WT GC-B (WT) or Tyr^808^ mutants (Y808F, Y808A, Y808E, Y808S and Y808W) were treated with 100 nM S1P, stimulated with 100 nM CNP for 5 min, and their cGMP production was measured. Hatched bars, blank bars and filled bars indicate cGMP production in HeLa cells treated without S1P and stimulated without CNP, treated without S1P and stimulated with CNP, and treated with S1P and stimulated with CNP, respectively. $, cGMP production was less than detectable level. (**B**) HeLa cells transiently expressing Myc-tagged WT GC-B or its Tyr^808^ mutants were preincubated with vehicle (filled bars), 100 ng/ml PMA (blank bars) or 0.5 mM Na_3_VO_4_ (grey bars) for 30 min, and then stimulated with CNP as described above. (**C**) Expression levels of Myc-tagged WT GC-B and its mutants (GC-B) and βAct (β-actin) in HeLa cells estimated by immunoblotting. Data represents means±S.EM. *n*=3. *, *P*<0.05; **, *P*<0.01; ***, *P*<0.001; when the drug treatment reduced the value of cGMP production and ^#^, *P*<0.05; ^##^, *P*<0.01, when the drug treatment elevated the value of cGMP production.

I next examined whether any of the Tyr^808^ mutations affected the suppression of GC activity by well-characterized GC-B inhibitors, such as S1P, PMA and Na_3_VO_4_. As demonstrated above, S1P-dependent suppression of GC-B activity was unaffected by the Y808F mutation, nor was it affected by the Y808W mutation (Figure 4A). However, the Y808A, Y808E and Y808S mutants were far less sensitive to S1P treatment, with the Y808E mutant showing the most dramatic resistance to inhibition (Figure 4A).

Figure 4(B) shows how the Tyr^808^ mutations affect GC-B inhibition by PMA and Na_3_VO_4_. PMA, an activator of PKC, is the most potent suppressor of GC-B activity currently known [21,24]. Na_3_VO_4_ [19], a non-specific tyrosine phosphatase inhibitor, also potently suppresses GC-B activity, by a mechanism believed to be similar to that of growth factor-mediated suppression of activity [19]. cGMP production by WT GC-B and the Y808F and Y808W mutants was suppressed to near-basal levels by PMA, whereas suppression of the Y808A mutant was greatly attenuated. Surprisingly, PMA treatment enhanced cGMP production in cells expressing the Y808E and Y808S mutants. Na_3_VO_4_ inhibited the activities of WT GC-B, as well as those of the Y808F, Y808A and Y808W mutants, but failed to significantly alter the activities of the Y808E and Y808S mutants.

### Hyperactive mutants exhibit near-maximum GC-B activity

In intact cells and membranes, the activities of membrane-bound GCs are tightly regulated by their extracellular ligands. However, once these GCs are solubilized with detergents (e.g., Triton X-100) in the presence of Mn^2+^, they exhibit maximal cyclase activities, which are not further increased in the presence of ligands [31]. To test the effects of the Tyr^808^ mutations on this ligand-independent activity of GC-B, I expressed the mutants in HeLa cells, prepared membrane fractions and measured CNP-dependent and Triton X-100/Mn^2+^-dependent GC activities of these fractions (Table 1). Consistent with the data shown above, membranes expressing WT GC-B and the Y808W mutant displayed similar CNP-stimulated cGMP production, whereas CNP-stimulated activities of membranes expressing the Y808F, Y808A, Y808E and Y808S mutants were more than 10-fold higher than WT. In contrast, Triton X-100/Mn^2+^ activities of WT and mutant GC-Bs were similar. Thus, the Tyr^808^ mutations elicited striking elevations of GC-B activity in the context of intact cells and membranes, without markedly altering the maximal catalytic potential of the cyclase.

**Table 1:** CNP- and TX-100/Mn^2+^-dependent cGMP production in WT GC-B and Tyr^808^ mutants Units are pmol/mg protein.

Type of GC-B	Basal (A)	CNP-dependent (B)	TX-100/Mn^2+^-dependent (C)	Ratio (B/C×100)
WT	1.4±0.3	6.0±0.2	310.1±0.5	1.9
Y808F	20.1±0.2	120.3±7.9	668.2±37.5	18.0
Y808A	25.4±3.4	171.4±33.9	552.1±64.4	31.0
Y808E	54.5±11.0	63.2±8.8	221.1±6.4	28.5
Y808S	31.6±8.3	71.5±8.0	117.5±7.7	60.8
Y808W	8.5±0.5	15.8±0.2	290.4±8.1	5.4

### Phosphorylation is not essential for GC activity of Y808E

**Figure 5:**
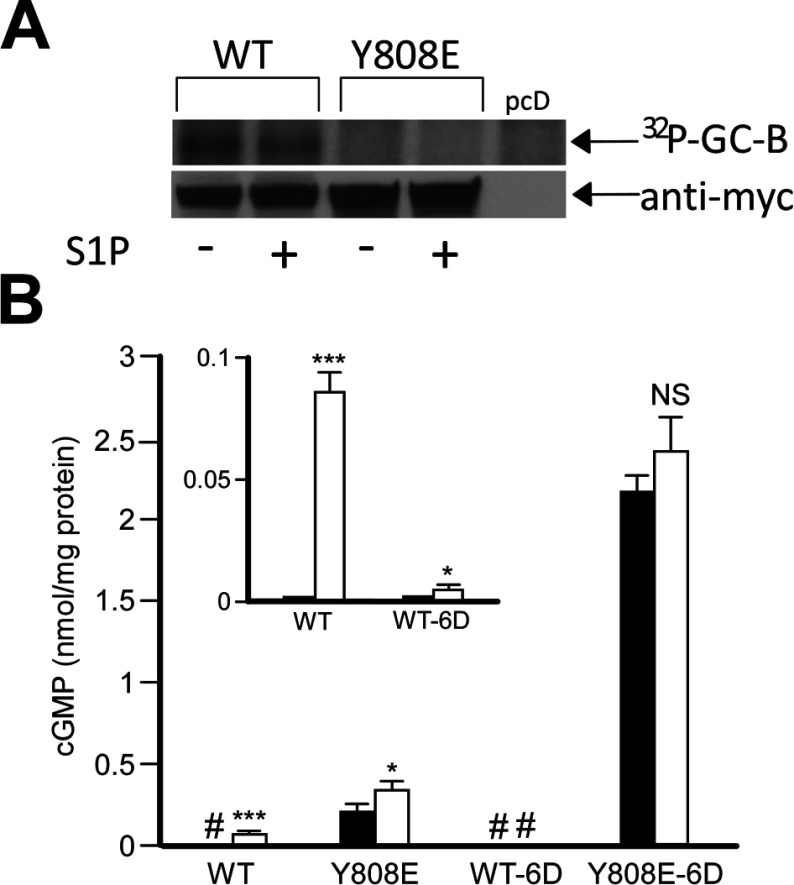
Phosphorylation levels (A), and effect of phosphomimetic mutation in the hyperphosphorylation sites on GC activity (B) in WT GC-B and its Y808E mutant (**A**) HeLa cells transiently expressing Myc-tagged WT GC-B (WT) or Y808E mutant (Y808E), or empty vector (pcD) were cultured with the medium containing ^32^P-orthophosphate and stimulated with 1 μM S1P for 30 min. The cells were then lysed with RIPA buffer, and immunoprecipitation was performed as described in ‘Experimental Procedures’. Upper panel shows phosphorylated bands detected by autoradiography, and panel shows WT Myc-GC-B and its Y808E mutant bands in HeLa cells probed with anti-myc antibody, and visualized with HRP (horseradish peroxidase)-conjugated secondary antibody and ECL system. The data represent one of three independent experiments. (**B**) HeLa cells transiently expressing Myc-tagged WT GC-B (WT), and Y808E, WT-6D and Y808E-6D mutants were cultured in the absence (filled bars) or presence (blank bars) of 100 nM CNP for 5 min, and their cGMP production was measured. #, see inset. Data represent means±S.E.M, *n*=3. *, *P*<0.05; ***, *P*<0.001.

As discussed above, GC-B is constitutively phosphorylated and both its homologous and heterologous desensitization is accompanied by its dephosphorylation [25]. Moreover, mutation of phosphorylated serines Ser^523^ and Ser^526^ to alanine or glutamine greatly reduced GC-B activity [26]. These observations have led to the widespread supposition that phosphorylation is an absolute requirement for GC-B activation. To examine the effect of S1P on the phosphorylation of the hyperactive GC-B mutants, I metabolically labelled HeLa cells expressing myc-tagged WT or Y808E GC-B with radioactive phosphate, stimulated cells with S1P and immunoprecipitated the receptors using an anti-myc antibody. As expected, S1P treatment resulted in 41±6% reduction (*P*< 0.01) of ^32^P_i_ incorporation level into WT GC-B (Figure 5A, left), which was consistent with a previous report [26]. In contrast, I was unable to detect phosphorylation of the Y808E mutant (Figure 5A, right).

I next introduced aspartic acid mutations (6D mutation) into all six potential phosphorylation sites in the juxtamembrane portion of both WT GC-B (WT GC-B-6D) and the Y808E mutant (Y808E-6D), and measured cGMP production in cells expressing these constructs, in the absence or presence of CNP (Figure 5B). Consistent with the previous report about GC-B having six glutamic acid mutations in the juxtamembrane phosphorylation sites [22], WT-GC-B-6D mutant exhibited greatly reduced cGMP production, which was slightly elevated upon CNP stimulation (Figure 5B, inset). Unexpectedly, Y808E-6D mutant produced remarkably higher amounts of cGMP in the absence of CNP, than did Y808E, although no significant elevation of CNP-induced cGMP production was observed (Figure 5B).

## DISCUSSION

**Figure 6:**
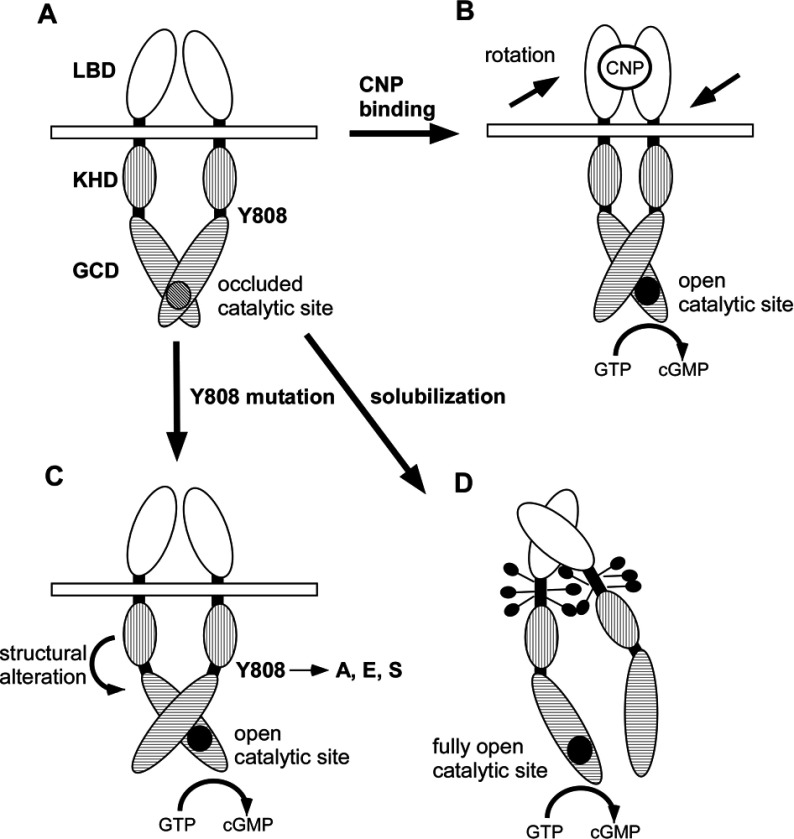
Hypothetical mechanism of (A) resting state, (B) ligand-dependent, (C) detergent-dependent and (D) mutation-dependent activation of GC-B (**A**) One of two catalytic sites of GC-B is occluded by another subunit of GC-B dimers. GTP is prohibited access to the catalytic sites. (**B**) Two subunits of the LBD dimer undergo re-orientation upon CNP binding, which is followed by further re-orientation of the intracellular domains to open the catalytic sites. GTP is then allowed to access the open catalytic sites to be converted to cGMP. (**C**) Tyr^808^ mutations induce structural alteration of the intracellular domain, which results in opening the catalytic site. (**D**) Treatment of GC-B with detergent perturbs orientation of two dimers and renders the catalytic sites open fully. LBD, ligand binding domain; CNP, C-type natriuretic peptide; KHD, kinase homology domain and GCD, guanylyl cyclase (catalytic) domain. Inactive catalytic site is shown with a diagonally striped circle, and active catalytic sites are shown with filled circles.

In this study I demonstrated that Tyr^808^ plays a critical role in the regulation of the enzymatic activity of GC-B. Mutations of Tyr^808^ resulted in a wide range of effects on cyclase activity, depending on the nature of the substituting residue (Figure 3). In addition, the fact that mutation of a single amino acid residue in GC-B results in catalytic hyperactivation points to an auto-inhibitory mechanism that regulates the activity of WT GC-B. Although the intracellular crystal structure of membrane-bound GC has not been solved, the extracellular crystal structure of hormone-bound ANP receptor [12] gives us clues to understand the auto-inhibition mechanism of GC-B. One probable auto-inhibition mechanism is that one of intracellular domain of GC-B dimer occludes another GC catalytic site, thereby limiting access of GTP to the catalytic site (Figure 6). As mentioned above, the two subunits of the LBD dimer undergo twisted motion, which also alters the orientation between the two intracellular domains to open their catalytic sites upon CNP binding (Figure 6B). This auto-inhibitory mechanism can be circumvented by replacement of the native residue, Tyr^808^, with a smaller (alanine or serine) or charged (glutamic acid) amino acid, probably because these mutations alter the orientation of GC domain to KHD, allowing GTP to access the catalytic site with less steric hindrance (Figure 6C). This auto-inhibitory model is likely to be relevant to the well-established observation that GC activity reaches to maximum upon solubilization with a detergent. Perhaps the proper arrangement of GC-B dimer is perturbed by the solubilization, and this perturbation may render the catalytic site open fully and producing cGMP with maximum activity (Figure 6D).

An unexpected consequence of the Y808A, Y808E and Y808E mutations was the markedly reduced suppression of GC-B activity by S1P, PMA and orthovanadate (Figure 4B). Indeed, GC activity was actually enhanced in the Y808E and Y808S mutants upon treatment with PMA, suggesting that PKC (or another target of PMA) regulates GC-B in a complex fashion, exerting both inhibitory and excitatory influences. Based on my model shown in Figure 6, I assume that the PKC or another target of PMA may alter the intracellular structure of GC-B, resulting in an unfavourable for WT GC-B to open the catalytic site but favourable for the Y808E and Y808S mutants.

Another unexpected outcome of this study was the finding that phosphorylation is apparently not necessary for expression of GC activity by theY808E mutant (Figure 5). This hyperactive mutant failed to incorporate radioactive phosphate in cells over a 6 h labelling period (Figure 5A), indicating that the Y808E mutant exhibits strong GC activity in cells without phosphorylation. Furthermore, the Y808E mutant carrying six aspartic acid mutations at the juxtamembrane phosphorylation motif exhibited much higher GC activity than that of Y808E, even though WT-GC-B containing the same six aspartic acid mutations exhibited only trace level of GC activity. Future structural analyses will elucidate the mechanism to clarify these results.

**Figure 7:**
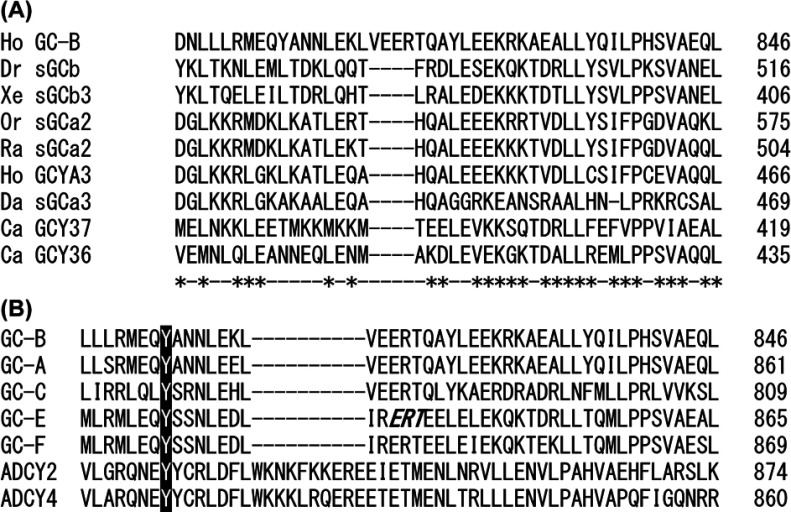
Alignment of amino acid sequences of human GC-B with HNOBA domain of soluble GCs (A), and with membrane bound GCs and ADCYs (B) (**A**) GC-B (amino acids 799–846) was aligned with a partial fragment of the HNOBA domain of soluble GCs of various species. Asterisks show amino acid residues of GC-B having significant similarity to soluble GCs. Ho, *Homo sapiens*; Dr, *Drosophila melanogaster*; Xe, *Xenopus laevis*, Or, *Oryzias latipes*; Ra, *Rattus norvegicus*; Da, *Danio rerio*; Ca, *Caenorhabditis elegans*. (**B**) Partial amino acid sequence of human GC-B including Tyr^808^ was aligned with those of HNOBA domain of all membrane-bound GCs, ADCY2 and ADCY4, all of which have tyrosine residue at the position equivalent to Tyr^808^ of GC-B. The alignment was constructed using the NCBI BLASTP amino acid sequence homology search engine. Highlighted residues show Tyr^808^ of GC-B and its equivalent tyrosine residues in other cyclases. Italic shows Arg^838^ of GC-E, and mutation of which has been linked to autosomal dominant inherited cone degeneration and cone–rod degeneration.

Based on NCBI (National Center for Biotechnology Information) Conserved Domain Database [32], Tyr^808^ is located in a niche domain between the KHD and GC domains and thus would not be expected to affect the structure of these domains directly. Interestingly, the niche domain containing Tyr^808^ shows sequence similarity to a partial fragment of HNOBA (haem nitric oxide binding associated) domain, which is found in soluble GCs and bacterial haem-binding protein kinases [33,34] (Figure 7A). HNOBAs consist of an N-terminal core subdomain having interspersed α-helices and β-strands, followed by an extended C-terminal α-helix, termed the helical linker region, which has the potential to form coiled-coils and, hence, may be involved in dimerization. Based on the sequence alignment (Figure 6A) and on secondary structure predictions of Iyer et al. [34], Tyr^808^ is located within the helical linker region. Mutations of Tyr^808^ may alter the dimerization state of GC-B and thereby control its catalytic activity, which fits our hypothesis shown in Figure 6.

All membrane-bound GCs, as well as adenylyl cyclases 2 and 4, have tyrosine at the position corresponding to residue 808 of GC-B (Figure 6B). Therefore it may be possible to generate a variety of cyclases with enhanced activity by substituting the Tyr^808^-equivalent tyrosine residue with alanine or phenylalanine, and of obtaining constitutively active cyclases by replacing that residue with serine or glutamic acid. Interestingly, mutations in Glu^837^, Arg^838^ and Thr^839^ of GC-E (shown in bold italics in Figure 7B), which are within 12 amino acids of the tyrosine residue equivalent to Tyr^808^ in GC-B, have been linked to dominant cone–rod dystrophy [35–38]. These three residues of GC-E are also conserved among all membrane-bound GCs. My constitutively active mutants may eventually prove to be valuable therapeutic tools, as GC-B activity has been shown to be protective against several disorders. For example, Yasoda et al. [39] showed that tissue-specific overexpression of CNP and activation of GC-B counteracts dwarfism in a mouse model of achondroplasia containing a hyperactive mutation of FGFR 3 (fibroblast growth factor receptor 3). Introduction of those constitutively active GC-B mutants may mimic the overexpressing CNP-induced GC-B activation in chondrocytes and is likely to suppress FGFR3-dependent achondroplasia.

Due to its location between the KHD and the GC domain, scant attention has been paid to the potential biochemical significance of Tyr^808^ or its neighbouring residues. Current models suggest that membrane-bound GCs are present in an auto-inhibited conformation in unstimulated cells, and that binding of natriuretic peptides to their extracellular domains releases this inhibitory constraint. Mechanisms that underlie GC auto-inhibition have remained elusive, primarily because three-dimensional structures of the intracellular domains of membrane-bound GCs have not yet been solved. My results suggest that Tyr^808^ contributes to the auto-inhibition of GC-B, and raises the possibility that binding of CNP to the receptor induces a conformational change that displaces this residue from its inhibitory orientation.
